# Auxin Metabolism Is Involved in Fruit Set and Early Fruit Development in the Parthenocarpic Tomato “R35-P”

**DOI:** 10.3389/fpls.2021.671713

**Published:** 2021-08-02

**Authors:** Shaoli Zhang, Xin Gu, Jingcheng Shao, Zhifeng Hu, Wencai Yang, Liping Wang, Hongyan Su, Luying Zhu

**Affiliations:** ^1^Key Laboratory of Molecular Module-Based Breeding of High Yield and Abiotic Resistant Plants in Universities of Shandong (Ludong University), College of Agriculture, Ludong University, Yantai, China; ^2^Institute of Vegetable, Gansu Academy of Agricultural Science, Lanzhou, China; ^3^College of Horticulture, China Agricultural University, Beijing, China; ^4^Agricultural and Rural Bureau of Shouguang, Shouguang, China

**Keywords:** fruit set, endogenous hormones, transcriptome, parthenocarpy, tomato

## Abstract

Parthenocarpic tomato can set fruit and develop without pollination and exogenous hormone treatments under unfavorable environmental conditions, which is beneficial to tomato production from late fall to early spring in greenhouses. In this study, the endogenous hormones in the ovaries of the parthenocarpic tomato line “R35-P” (stigma removed or self-pollination) and the non-parthenocarpic tomato line “R35-N” (self-pollination) at four stages between preanthesis and postanthesis investigated, using high-performance liquid chromatography-tandem mass spectrometry (HPLC-MS/MS). A nearly twofold IAA (indoleacetic acid) content was found in “R35-P” rather than in “R35-N” at −2 and 0 days after anthesis (DAA). Except at −2 DAA, a lower ABA (abscisic acid) content was observed in Pe (stigma removed in “R35-P”) compared to that in Ps (self-pollination in “R35-P”) or CK (self-pollination in “R35-N”). After pollination, although the content of GA_1_ (gibberellins acid 1) in CK increased, the levels of GAs (gibberellins acids) were notably low. At all four stages, a lower SA (salicylic acid) content was found in Ps and CK than in Pe, while the content and the change trend were similar in Ps and CK. The variation tendencies of JA (jasmonic acid) varied among Pe, Ps, and CK at the studied periods. Furthermore, KEGG (Kyoto Encyclopedia of Genes and Genomes) enrichment analyses of transcriptomic data identified 175 differentially expressed genes (DEGs) related to plant hormone signal transduction, including 63 auxin-related genes, 27 abscisic acid-related genes, 22 ethylene-related genes, 16 cytokinin-related genes, 16 salicylic acid-related genes, 14 brassinosteroid-related genes, 13 jasmonic acid-related genes, and 4 gibberellin-related genes at −2 DAA and 0 DAA. Our results suggest that the fate of a fruit set or degeneration occurred before anthesis in tomato. Auxins, whose levels were independent of pollination and fertilization, play prominent roles in controlling a fruit set in “R35-P,” and other hormones are integrated in a synergistic or antagonistic way.

## Introduction

The off-season cultivation of tomato usually involves treatment with exogenous hormone regulators to stabilize a fruit set, which is a common practice in greenhouses with little wind and insects. However, the application of synthetic hormones results in the development of malformed fruits (Martinelli et al., 2009; Takisawa et al., 2019). The use of bumblebees can avoid these drawbacks but requires the maintenance of optimal temperatures to keep them active (Takisawa et al., 2017). All of these agricultural practices increase financial and labor costs for farmers.

Parthenocarpy can overcome these problems by converting the ovary into a developing fruit without pollination and exogenous hormone application, thereby producing seedless fruit. Currently, for highly desired agronomic traits, many vegetable crops, such as tomato (Gorguet et al., 2005, 2008; Takisawa et al., 2017, 2020), cucumber (Wu et al., 2016; Li et al., 2017), and eggplant (Du et al., 2016; Chen et al., 2017) can naturally produce parthenocarpic fruit. The use of parthenocarpic cultivars is considered to be the most cost-effective solution for a stable fruit set under suboptimal environmental conditions. The trait of parthenocarpy has increasingly attracted the interest of scholars and has become a popular topic for research.

Natural parthenocarpy in tomato has been widely studied for its potential use. To date, six parthenocarpic tomato resources and nine different parthenocarpic genes have been reported. “Soressi” and “Montfavet191” carry the *pat* gene (Soressi, 1975; Philouze and Pecaut, 1986), “Severianin” carries the *pat-2* gene (Philouze and Maisonneuve, 1978), “RP75/59” carries the *pat3/pat4* gene (Philouze, 1989), “IL5-1” carries the *pat4.1/pat5.1* gene (Finkers et al., 2007; Gorguet et al., 2008), “IVT-line1” carries the *pat4.2/pat9.1* gene (Zijlstra, 1985; Gorguet et al., 2008), and “MPK-1” carries the *pat-k* gene (Takisawa et al., 2017, 2020). The parthenocarpic genes *pat-2* and *pat-k* have been cloned. The *pat-2* gene is located on chromosome 4 and encodes a zinc-finger homeodomain (ZHD) protein, whereas the *pat-k* gene is located on chromosome 1 and encodes an E-class MADS-box gene, SlAGAMOUSLIKE6 (*SLAGL6*) (Takisawa et al., 2018, 2020). The *Pat* locus was mapped on the long arm of tomato chromosome 3, and the two SCAR markers SSR300 and SSR601 were located distally from the *Pat* locus at 7.5 and 3.7 cM, respectively (Beraldi et al., 2004). The *pat4.1* and *pat4.2* were likely allelic and located on chromosome 4, whose potential candidate gene was *ARF8* (Gorguet et al., 2008). The *pat5.1* and *pat9.1* were located on chromosomes 5 and 9, respectively (Gorguet et al., 2008). The *pat3/pat4* genes were located on chromosome 1 L site 152 and chromosome 6 L site 34 (Vardy et al., 1989).

It has been shown that parthenocarpic tomato is mainly controlled by endogenetic hormones. Hazra et al. (2010) showed that the content of auxin in “Oregon Pride” (*pat-2*) was 10.48 times higher than that of the control at 3 days before anthesis. Dai (1998) pointed out that the IAA content was significantly higher in parthenocarpic tomato than in non-parthenocarpic tomato from 2 days before anthesis to 4 days after anthesis. Furthermore, artificially increasing the endogenous auxin levels of the ovary by introducing *DefH9-iaaM* or *TIR1* or by inhibiting *AUX/IAA*, *ARFs*, or *Aucsia* could also stimulate parthenocarpy in tomato (Pandolfini et al., 2002; Rotino et al., 2005; Wang et al., 2005; Goetz et al., 2007; de Jong et al., 2009; Molesini et al., 2009; Mazzucato et al., 2015). Gibberellins have also been regarded as key regulators in the parthenocarpic fruit set and development of tomato (Gorguet et al., 2005; Serrani et al., 2008). Higher GA levels have been found in the ovaries of natural parthenocarpic tomato *pat*, *pat-2*, and *pat3/pat4* mutants, and endogenous GA levels increase in tomato ovaries, following pollination (Mariotti et al., 2011; and literature cited therein). DELLA proteins are plant nuclear factors that restrain growth and proliferation in response to hormonal signals, and they have been characterized as repressors of gibberellin signaling (Marti et al., 2007). Silencing *DELLA* induces fertilization-independent fruit growth in tomato (Marti et al., 2007). It is generally recognized that the parthenocarpy of these tomato resources is regulated by the coordination of endogenetic hormones, especially auxins and gibberellins (GAs) (Serrani et al., 2008; Pascual et al., 2009; de Jong et al., 2011; Mignolli et al., 2015). Although an increasing number of studies on hormones have been conducted, the mechanism governing parthenocarpy has not been fully elucidated to date.

The parthenocarpy of tomato “R35” is controlled by a recessive gene (Wang et al., 2008), but little is known about its physiological basis and molecular mechanism. In this study, to improve the parthenocarpic rate and growth potential of “R35,” we developed a new line, “R35-P,” by self-crossing and selection. During this period, we found a non-parthenocarpic plant, “R35-N,” and we cultivated it by self-pollination and purification. Biochemical and molecular mechanisms of ovary development in “R35-P” were elucidated by combining the measurement of endogenous hormones with the analysis of DEGs related to plant hormones based on transcriptomic data. This study will not only be helpful to understand the molecular mechanisms by which hormones regulate the fruit set of “R35-P” but may also establish a theoretical foundation for the creation of genetic parthenocarpy in tomato by genetic engineering.

## Materials and Methods

### Plant Materials and Treatment

Parthenocarpic and non-parthenocarpic tomato lines were used in this study. Both lines were isolated from the parthenocarpic tomato line “R35” (Wang et al., 2008) by selfing and selection.

**FIGURE 1:**
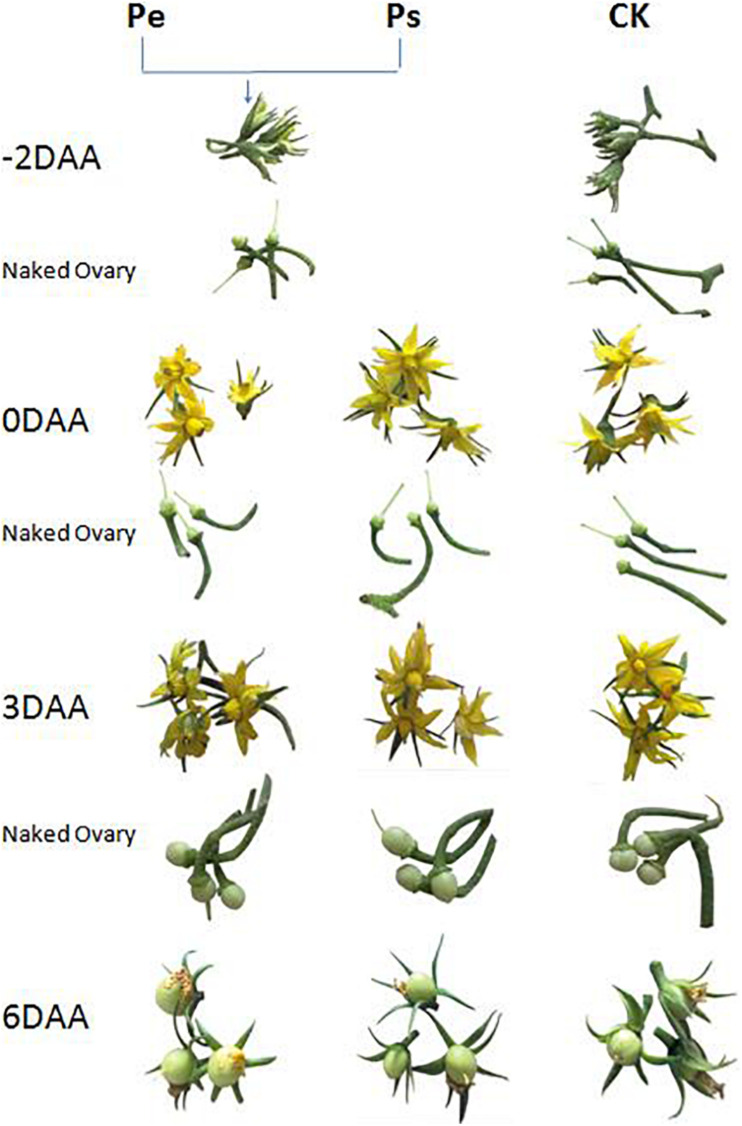
Morphological changes of flowers and ovaries during the early development stages in Pe (stigma removed in “R35-P”), Ps (self-pollination in “R35-P”), and CK (self-pollination in “R35-N”).

The stigmas of “R35-P” buds were removed 2 days before anthesis to prevent self-pollination, and the flowers emasculated were denoted Pe; “R35-P” was self-pollinated at anthesis and denoted Ps; and “R35-N” was self-pollinated at anthesis and denoted CK. The ovaries of Pe, Ps, and CK were tagged and collected at −2, 0, 3, and 6 days after anthesis (DAA) (Figure 1), frozen in liquid N_2_, and stored at −80°C until needed for hormone quantification or RNA-seq analysis.

### Measurement of Endogenous Hormone Levels

For endogenous plant hormone analyses, the naked ovaries, receptacles, and intact fruit petioles of Pe, Ps, and CK at −2, 0, 3, and 6 DAA were collected (Figure 1). About 1.5 g per bulked sample was used, which did not take from the fixed single plant but from the line consisting of more than 100 individual plants.

The levels of indole-3-acetic acid (IAA), gibberellin (GA_1_,_3_,_4_,_5_,_7_), abscisic acid (ABA), jasmonic acid (JA), and salicylic (SA) were measured at Zoonbio Biotechnology Co., Ltd. (Nanjing, China). Approximately, 1.0-g ovaries were ground in a precooled mortar that contained a 10-ml extraction buffer composed of isopropanol/hydrochloric acid. The extract with 8-μl internal standard (1 μg/ml) (D-IAA, D-ABA, D-SA, D-GA, and 2HJA) added was shaken at 4°C for 30 min. Then, 20-ml dichloromethane was added, and the sample was shaken at 4°C for 30 min and centrifuged at 13,000 rpm for 5 min at 4°C. The lower, organic phase was extracted, which was dried under N_2_ and dissolved in 400-μl methanol (0.1% methane acid) and filtered with a 0.22-μm filter membrane. The purified product was then subjected to high-performance liquid chromatography-tandem mass spectrometry (HPLC-MS/MS) analysis. HPLC analysis was performed, using a poroshell 120 SB-C18 (Agilent Technologies) column (2.1 × 150 mm; 2.7 μm). The mobile phase A solvents consisted of methanol/0.1% methanoic acid, and the mobile phase B solvents consisted of ultrapure water/0.1% methanoic acid. The injection volume was 2 μl. MS conditions were as follows: the spray voltage was 4,500 V; the pressure of the air curtain, nebulizer, and aux gas were 15, 65, and 70 psi, respectively, and the atomizing temperature was 400°C.

### RNA Extraction and RNA-Seq Analysis

The RNA preparation, cDNA libraries construction, and Illumina sequencing were performed in Novogene (Beijing, China). Total RNA was extracted from freshly frozen naked ovaries (with receptacles and fruit pedicels), three biological replicates per sample, using the Trizol reagent (Invitrogen, Carlsbad, CA, United States) and purified using the RNeasy Plant Mini kit (Qiagen, Valencia, CA, United States). About 0.5 g ovaries were sampled per biological repeat sample for RNA-seq, and 0.2 mg of which was used for RNA extraction. RNA integrity and purity were assessed by agarose GEL electrophoresis and Agilent 2100 bioanalyzer. Then, mRNA was purified from total RNA, using poly-T oligo-attached magnetic beads and was interrupted into 250–300 bp short segments. The effective concentration of RNA library was higher than 3 nM. First strand cDNA was synthesized, using random oligonucleotide primers. Second strand cDNA synthesis was subsequently performed, using DNA Polymerase I and RNase H. After end repair, the adaptor was added to the cDNA with T_4_ DNA ligase. The appropriate fragment ligated with adaptor was selected by AMPure XP beads as a template for PCR amplification. The cDNA library products were sequenced by the Illumina HiSeq 2000 platform.

The raw data obtained by sequencing were quality controlled, which mainly included removal of the reads with adapters, removal of the reads with a ratio of N more than 0.002 (N denotes unidentifiable base information) and removal of the paired reads when the number of low-mass bases in a single-ended read was more than 50% of the read length. Then, the clean reads got by examination of the sequencing error rate and GC content distribution. The clean reads were compared and analyzed with the Hisat2 software^1^ and then were spliced. By comparing with the tomato reference genome (Heinz 1706), the genes expression of RNA-seq were obtained, which were expressed by FPKM (fragments per kilobase of transcript sequence per millions base pairs sequenced).

The transcription profiles of the unpollinated and pollinated ovaries at −2, 0, 3, and 6 DAA of Pe, Ps, and CK were compared, with three biological replicates per sample.

### GO Enrichment and KEGG Pathway Analyses

The differentially expressed genes (DEGs) were identified through pairwise comparison between the ovaries of Pe, Ps, and CK libraries. The filtered DEGs were subjected to GO enrichment analysis with the GOseq R package, and the pathways of these DEGs were annotated with the BLASTALL program against the KEGG (the Kyoto Encyclopedia of Genes and Genomes) database, respectively. Categories with Padj < 0.05 were considered significantly enriched.

### Quantitative Real-Time PCR Analysis

Ten genes were selected randomly for RNA-seq data validation, using qRT-PCR. Total RNA was extracted from ovaries samples of tomato as described above and was reverse-transcribed in a 20-μl reaction system with a cDNA synthesis kit (RR047A, TaKaRa, Dalian, China) according to the instructions of the manufacturers. Then, the products were subjected to 10-fold dilution and used in subsequent qPCR. qPCR was prepared in reactions containing 10-μl 2 × SYBR Green real-time PCR Master Mix (Takara, Dalian, China), 3-μl diluted first-strand cDNA,0.8 μl each PCR primer (10 μM), and distilled water to a 20-μl volume. The amplification procedure was set as follows: 95°C for 30 s, then 40 cycles of 95°C for 5 s, 60°C for 34 s, and 72°C for 45 s. The qRT-PCR was performed in three replicates per sample. Tomato *actin* gene (*Solyc03g078400*) was used as an internal control. The 2^–ΔΔCt^ method (Livak and Schmittgen, 2001) was used for the calculation of relative expression levels of target genes. Primers used for qPCR assays are listed in Supplementary Table 2.

### Statistical Analysis

All data were presented as the means ± standard errors of three biological replicate samples. The statistical significance of the pairwise comparison among the three groups was tested by the two-way ANOVA method, and *P* < 0.05 was indicated.

## Results

### Performance of “R35-P” and “R35-N”

**FIGURE 2:**
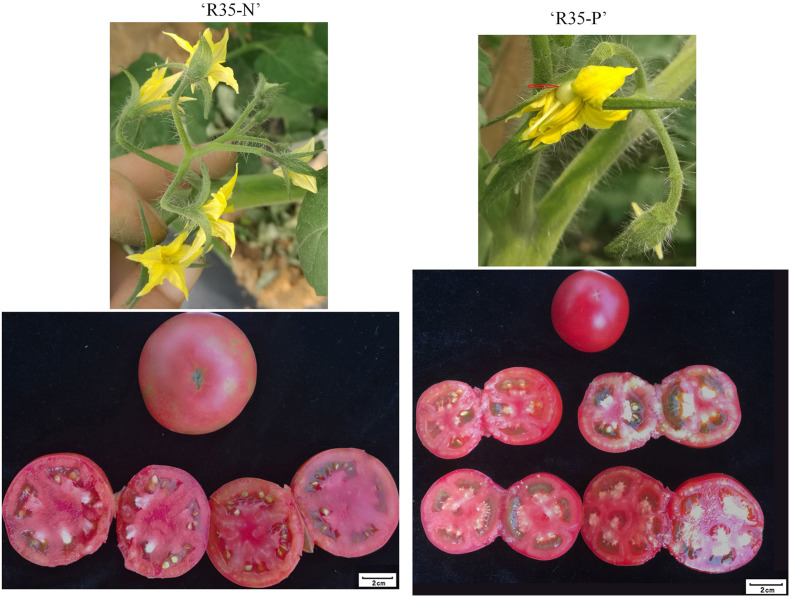
The phenotypes of flowers and tomato fruits of “R35-N” and “R35-P.” The seeded fruits of “R35-N” and “R35-P” obtained by natural pollination at an open field, while the seedless fruits of “R35-P” that obtained flowers emasculation carried 2 days before anthesis.

“R35-P” shows environmentally stable facultative parthenocarpy under different conditions, and displays accelerated ovary expansion before or at anthesis occasionally (Figure 2). It belongs to the infinite growth type, medium growth. The seeded and seedless fruits (parthenocarpic fruits with jelly) of “R35-P” were similar in size on the same plant (Figure 2), and single fruit weight is about 80 g, with 3–4 locules (the average ratio of polar and equatorial diameter is about 4/4.3). The fruits are pink and round. The percentage of a parthenocarpic fruit set is higher than 80%, if the stigmas of buds for the first three spikelet flowers in “R35-P” were removed 2 days before anthesis. And it can produce normal seeded fruits or seedless fruits (encounter unfavorable weather conditions) naturally when planted in an open field.

Unpollinated fruits of line “R35-N” will abort, and self-pollinated fruits are slightly larger than the seeded/seedless fruits of “R35-P”; and single fruit weight is about 100 g with 4–5 locules (the average ratio of polar and equatorial diameter is about 5/5.1) (Figure 2). “R35-N” has a genetic background similar to that of “R35-P.”

### Endogenous Hormone Levels in “R35-P” and “R35-N”

**FIGURE 3:**
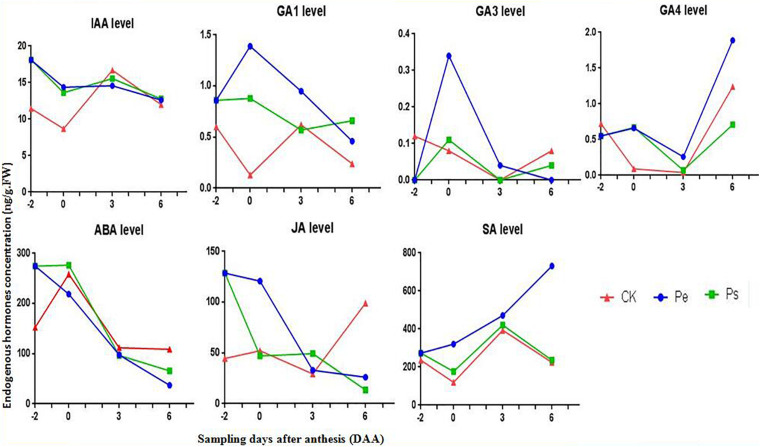
Comparison of the phytohormone levels (IAA, ABA, GA_1_,_3_,_4_, SA, and JA) between Pe (stigma removed in “R35-P”), Ps (self-pollination in “R35-P”), and CK (self-pollination in “R35-N”) at −2, 0, 3, 6 days after anthesis (DAA).

The levels of endogenous auxins (IAA), gibberellins (GA_1_,_3_,_4_,_5_,_7_), abscisic acid (ABA), jasmonic acid (JA), and salicylic acid (SA) in naked ovaries of Pe, Ps, and CK were determined at four periods (−2, 0, 3, and 6 DAA) (Figure 3).

A significantly higher IAA content (nearly twofold) was found in “R35-P” regardless of whether it was pollinated than in CK at −2 and 0 DAA, and a similar content was found at 3 and 6 DAA. The levels of IAA were almost identical in Pe and Ps, and the changes in the IAA level were similar in Pe, Ps, and CK at all four stages. This result indicated that IAA was associated with genotype (parthenocarpic or not) but not with fruit setting development direction (seeded or seedless in parthenocarpic tomato); IAA was independent of pollination.

The levels of GA_1_, GA_3_ and GA_4_ were notably low, and GA_5_ and GA_7_ were under the detection limits at these stages. The variation tendencies of GA_1_, GA_3_, and GA_4_ were similar in Pe and Ps, contrary to CK, from −2 DAA to 0 DAA. The contents of GA_1_ and GA_3_ were significantly higher in Pe than in Ps, while GA_4_ was almost equal in Pe and Ps at 0 DAA. Additionally, only the content of GA_1_ in CK was increased, following pollination (0–3 DAA), while that in the other treatments was reduced.

The content and the change trend of SA were similar in Ps and CK, but the content of SA was lower in Ps and CK than in Pe at all four stages.

The content of JA was considerably higher in Pe and Ps than in CK at −2 DAA, and a pronounced difference was observed between Pe and Ps or CK at 0 DAA. The variation tendencies of JA varied among Pe, Ps, and CK at the studied periods.

Except at −2 DAA, the ABA content of Pe was lower than that of Ps and CK. The highest content appeared at 0 DAA. Following pollination (0–3 DAA), the ABA contents in CK and Ps were reduced.

### Analysis of RNA-Seq Data

Based on morphological observations and endogenous hormone level measurements, we found that the tomato ovaries were consistently triggered to set fruit before 3 DAA. Therefore, only the data of high-throughput RNA-seq of the samples at −2 and 0 DAA were used to analyze in this study. In total, 277 million reads, with approximately 5.5 million reads being obtained from each sample, were aligned with the reference genome (Supplementary Table 1).

**FIGURE 4:**
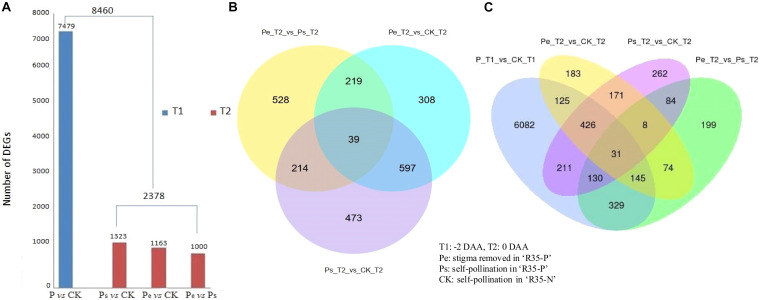
DEGs (differentially expressed genes) of the RNA-seq data. **(A)** The numbers of DEGs distribution (Padj < 0.05). **(B)** The Venn diagrams of 2,378 DEGs at 0 days after anthesis (DAA). **(C)** The Venn diagrams of 8,460 DEGs at −2 DAA and 0 DAA.

Based on the FPKM method, the transcript abundance of each gene was analyzed. Padj < 0.05 was used as the threshold to judge the significance of the differences in gene expression. A total of 8,460 differentially expressed genes (DEGs) were detected among Pe, Ps, and CK at −2 DAA and 0 DAA; of which, 7,479 DEGs were detected between P (Pe/Ps) and CK at −2 DAA, while 2,378 DEGs were identified among Pe, Ps, and CK at 0 DAA (Figure 4A). Among the DEGs identified at −2 DAA, 1,323 DEGs were found between Ps and CK, 1,163 were found between Pe and CK, and 1,000 were found between Pe and Ps. Detailed information on these common and specific DEGs was screened by Venn analysis (Figures 4B,C). A total of 39 DEGs were common to Pe, Ps, and CK at 0 DAA (Figure 4B), and 31 were common at −2 DAA and 0 DAA (Figure 4C).

**FIGURE 5:**
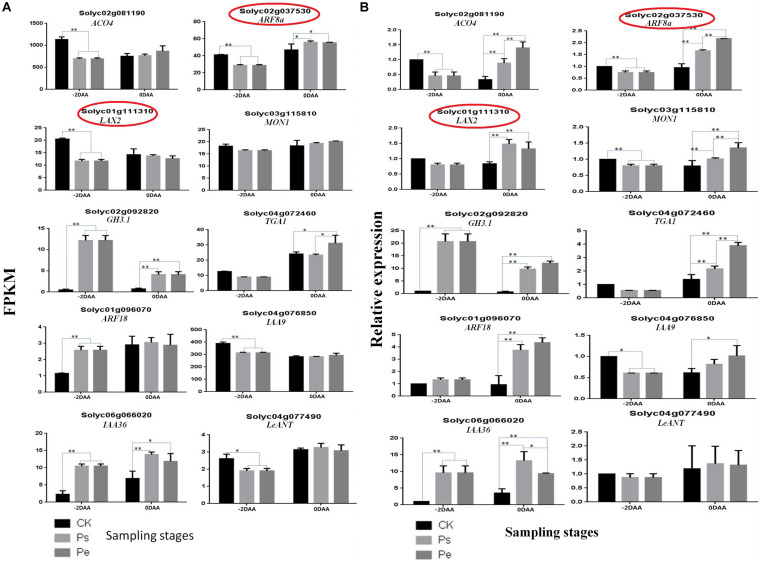
Validation of RNA-seq data by RT-PCR. **(A)** Expression levels of 10 DEGs were detected by RT-PCR, normalized based on the expression of the internal control gene, at −2 DAA (days after anthesis) and 0 DAA. **(B)** Corresponding genes expression were detected by RNA-seq based on the FPKM method (Padj < 0.05). The genes denoted by° had a different trend at 0 DAA between RT-PCR and RNA-seq. The bars represent standard errors of three replicates (*n* = 3). ^∗^*P* < *0.01*, ^∗∗^*P* < *0.001* by two-way ANOVA.

To further validate the reliability of the RNA-seq results, 10 genes (Supplementary Table 2) were randomly selected for a qRT-PCR comparison of their expression levels in the aforementioned samples at −2 DAA and 0 DAA. The results showed that most of the genes had a similar expression pattern between qRT-PCR and RNA-seq analyses (Figure 5), except that the expression of two genes (*Solyc01g111310* and *Solyc02g037530*) had different patterns at 0 DAA, indicating that the RNA-seq data were reliable and suitable for further analysis.

### Functional Enrichment Analysis of DEGs

The 7,479 DEGs (Supplementary Table 4) at −2 DAA and 2,378 DEGs (Supplementary Table 5) at 0 DAA among Pe, Ps, and CK identified by RNA-seq were subjected to GO and KEGG enrichment analyses.

**FIGURE 6:**
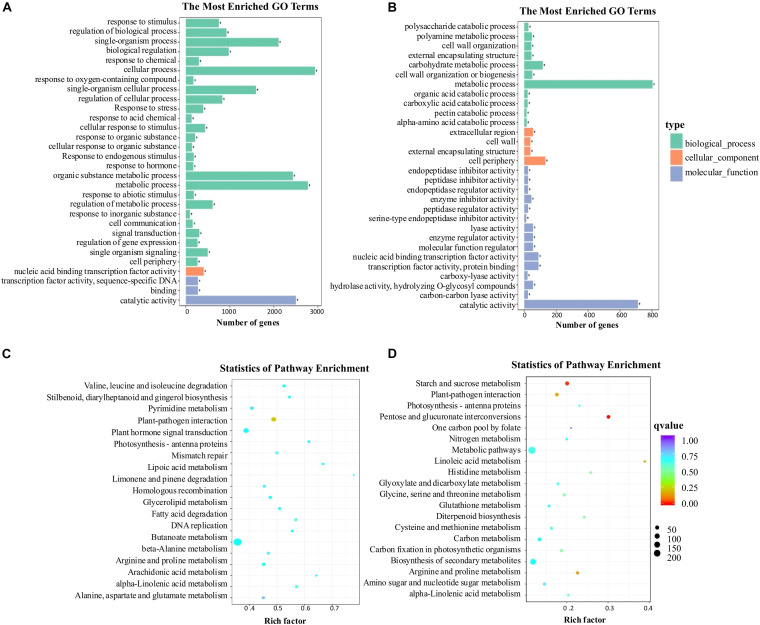
Functional categorization with the DEGs (differentially expressed genes). **(A)** GO analysis was performed with the DEGs at −2 DAA (days after anthesis). **(B)** GO analysis was performed with the DEGs at 0 DAA. **(C)** KEGG analysis was performed with the DEGs at −2 DAA. **(D)** KEGG analysis was performed with the DEGs at 0 DAA.

Gene ontology (GO) enrichment analysis provided all the GO terms that were significantly enriched for DEGs. The DEGs were assigned to 30 GO terms at −2 DAA and 0 DAA, which were summarized into three major GO categories: biological process (BP), cellular components (CC), and molecular function (MF) (Figures 6A,B). In the BP category, at −2 DAA, the largest number of DEGs was clustered into 26 groups, of which “cellular processes,” “metabolic processes,” “organic substance metabolic process” and “single-organism process” were dominant. The term “response to hormone” was also found in BP. Only one term, “cell periphery,” was found in the CC category at −2 DAA. Three terms were found in the MF category, with the most abundant DEGs being associated with “catalytic activity” at −2 DAA. Compared to the GO terms at −2 DAA, there were significant differences at 0 DAA. The number of groups in the BP category decreased from 26 to 11, and most DEGs were distributed in “metabolic processes,” while “response to hormone” disappeared. The number of groups in MF rose from 3 to 15, but “catalytic activity” was still the most abundant. The CC category had the fewest DEGs. The DEGs related to “metabolic processes” and “catalytic activity” were dominant at the two periods, suggesting that the ovaries were highly vigorous. To further identify the biological pathways that were active in parthenocarpic tomato, the DEGs were mapped to the reference pathways in the KEGG database (Figures 6C,D). At −2 DAA, the most significant pathways consisted of “biosynthesis of secondary metabolites,” “plant hormone signal transduction,” and “plant-pathogen interaction.” At 0 DAA, the DEGs were primarily distributed in “metabolic pathways,” “biosynthesis of secondary metabolites,” “starch and sucrose metabolism” pathways. As with GO enrichment, the “plant hormone signal transduction” pathway was only present at −2 DAA. Therefore, we inferred that hormone-related genes were actively expressed before anthesis to regulate a fruit set in tomato. Thus, we further analyzed plant hormone-related pathways and their gene expression patterns.

### Differentially Expressed Genes in Response to Phytohormones

**FIGURE 7:**
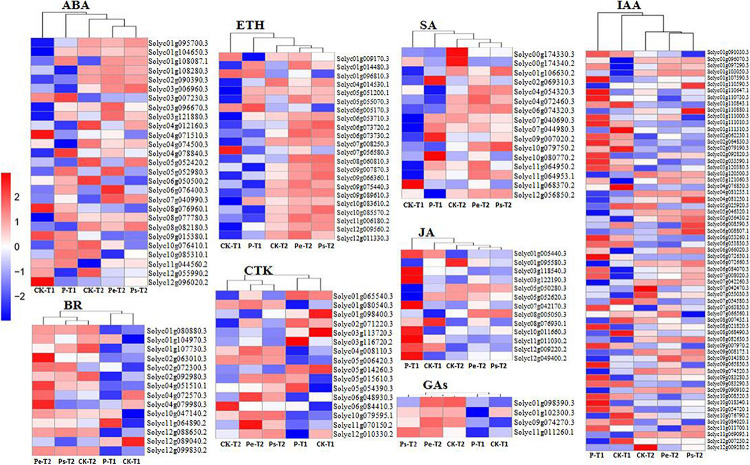
The heatmaps about hormones biological functions DEGs enriched by KEGG analysis. The FPKMs were homogenized by Z-score, which was a log based on multiple and folded changes with pairwise comparisons. The red and the blue colors indicate high and low transcript abundance.

To investigate whether changes in hormone levels were associated with changes in the activity of hormone metabolism genes, transcript levels of hormone metabolism genes were analyzed. KEGG enrichment analysis identified 175 DEGs (Supplementary Table 3) in the plant hormone signaling pathway, including IAA, CTK (cytokinin), GAs, ABA, ETH (ethylene), BR (brassinosteroid), JA, and SA, which were found at −2 DAA and 0 DAA, especially at −2 DAA. The differential expression of these genes in the studied samples is displayed in heatmaps (Figure 7). DEGs in Pe, Ps, and CK demonstrated similar gene expression patterns at the same period, and the gene expression profiles of Pe and Ps were clustered together, except for GAs at 0 DAA. Furthermore, there was a darker color and stronger contrast in the heatmaps for the DEGs in samples at −2 DAA than those at 0 DAA (Figure 7), which indicated that hormone-related genes were actively expressed before anthesis to regulate a fruit set in tomato. It was consistent with previous findings regarding GO and KEGG enrichment analysis.

The number of DEGs involved in different hormone metabolism pathways showed that the greatest number involved IAA followed by ABA, ETR, CTK, SA, and BR, and the fewest involved JA and GAs. The Auxin-signaling pathway, which is regarded as a key regulator of a fruit set in parthenocarpy, included 63 auxins-related genes. These genes encode members of the SAUR, AUX/IAA, GH3, AUX1, TIR1, and ARF protein families. Most of these genes are involved in auxin response genes, such as *SAUR* (covered 28 genes), *AUX/IAA* (covered 16 genes) and *ARF* (covered 5 genes). The functions of SAUR proteins are capable to modulate auxin synthesis and transport (Zhu et al., 2014). In this study, most *SAURs*, such as *Solyc01g110647.1*, *Solyc01g110730.3*, *Solyc03g033590.1, Solyc10g018340.1* and so on, showed upregulated expression in “R35-P” and downregulated expression in “R35-N” at −2 DAA (Figure 7). In the auxin-signaling components *AUX/IAAs* and *ARFs* family, *IAA1* (*Solyc09g083280.3*), *IAA2* (*Solyc06g084070.3*), *IAA3* (*Solyc09g065850.3*), *IAA16* (*Solyc01g097290.3*), *IAA19* (*Solyc03g120380.3*), *IAA26* (*Solyc03g121060.3*), *IAA29* (*Solyc08g021820.3*), *IAA35* (*Solyc07g008020.3*), *IAA36* (*Solyc06g066020.3*), *ARF1* (*solyc01g103050.3*), and *ARF18* (*Solyc01g096070.3*) showed upregulated expression in “R35-P” and downregulated expression in “R35-N,” while *IAA8* (*Solyc12g007230.2*), *IAA9* (*Solyc04g076850.3*), *IAA13* (*Solyc09g090910.2*), *IAA17* (*Solyc06g008590.3*), *IAA27* (*Solyc03g120500.3*), *ARF5* (*Solyc04g081235.1*), *ARF7* (*Solyc07g042260.3*), and *ARF9* (*Solyc08g082630.3*) were the opposite at −2 DAA (Figure 7). And the two libraries showed a slight difference among Pe, Ps, and CK, except the *IAA7* (*Solyc06g053830.3*) and *IAA14* (*Solyc09g083290.3*) showed different expression at 0 DAA (Figure 7). Validation by qRT-PCR showed that the expressions of auxin related genes *ARF18* (*Solyc01g096070.3*), *LAX2* (*Solyc01g111310.3*) and *IAA9* (*Solyc04g076850.3*) were extremely significant different among Pe, Ps and CK at −2 DAA, while which were similar expressed at 0DAA (Figure 5). The expressions of auxin-related genes *GH3.1* (*Solyc02g092820.3*) and *IAA36* (*Solyc06g066020.3*) were significantly or extremely significant different at −2 and 0 DAA among Pe, Ps, and CK (Figure 5). Twenty-seven related genes were found in the ABA-signaling pathway, which encodes members of the PYR/PYL, PP2C, SnPK2, and ABF protein families. The “ABA-PYR/PYL/RCAR-PP2C-SnRK2” signaling pathway of ABA may be suggested in tomato. Some *PP2C* genes (*Solyc03g121880.3*, *Solyc05g052980.3*, *Solyc06g076400.3*, *Solyc07g040990.3*) and *PYR/PYL* genes (*Solyc06g050500.2*, *Solyc08g076960.1*, *Solyc08g082180.3*, *Solyc09g015380.1*, *Solyc10g076410.1*, and *Solyc10g085310.1*) are greater expressed in “R35-P” than in “R35-N” at −2 DAA (Figure 7). In the ETH-signaling pathway, 22 related genes-encoding members of the ETR, CTR1, MPK6, EBF1/2, EIN3, ERF1/2, and EIN2 families were detected. Most of these genes mainly downregulated expressed, and more upregulated genes, such as *EIN3* (Solyc01g014480.3), *ERF1* (Solyc05g051200.1), *ETR* (Solyc05g055070.3), *MPK6* (Solyc06g005170.3), and *CTR1* (Solyc10g083610.2) were found in “R35-P” than in “R35-N” at −2 DAA (Figure 7). In the CTK-signaling pathway, 16 related genes-encoding members of the CRE1, AHP, B-ARR, and A-ARR families were detected. In the SA-signaling pathway, 16 related genes-encoding members of the NPR1, TGA, and PR-1 families were detected. In the BR-signaling pathway, 14 related genes-encoding members of the BSK, BZR1/2, CYCD3, BAK1, BRI1, and BIN2 families were detected. In the JA-signaling pathway, 13 related genes-encoding members of the JAR1, JAZ, MYC2, and COI1 families were detected. In the GA-signaling pathway, only four related genes-encoding members of the G1D1, DELLA, and TF families were detected.

## Discussion

The fruit set of plants largely depends on the biosynthesis and crosstalk of phytohormones. The activities of two plant growth hormones, auxins, and gibberellins are considered to be the main stimulus in the induction of a fruit set (Fos et al., 2000; Olimpieri et al., 2007; Serrani et al., 2008; de Jong et al., 2011; Mariotti et al., 2011; Mignolli et al., 2015; Tang et al., 2015).

In our study, the IAA content in the ovaries of CK was significantly lower than that in “R35-P” at preanthesis and anthesis, while it increased rapidly to the level of “R35-P” (pollinated or not), following pollination at postanthesis. This result was consistent with the findings of previous research on the parthenocarpic fruits of the *pat* mutant (Mapelli et al., 1978). The IAA content of ovaries in Ps, which did not increase following pollination, was almost equal to that in Pe at all-time points. Therefore, we suggested that the threshold concentration of IAA in the ovaries of “R35-P” was sufficient to promote a fruit set and did not require pollination stimulation. Additionally, the largest number of IAA-related DEGs was shown in hormone metabolism pathways at the transcript level. ARF and Aux/IAA proteins physically interact to regulate auxin response, are the best-characterized pates, and crucial in a triggering fruit set (Wang et al., 2005; Dorcey et al., 2009; Ruiu et al., 2015). Consistent with them, our results showed that some Aux/IAAs and ARFs, which accounted for 21/63 of the IAA-related DEGs, displayed dramatic shifts in their expressions between “R35-P” and “R35-N,” especially at −2 DAA. In our study, *IAA1*, *IAA2*, *IAA3*, *IAA16*, *IAA19*, *IAA26*, *IAA29*, *IAA35*, and *IAA36*, *ARF1*, *ARF9*, and *ARF18* upregulated expression in “R35-P” at −2 DAA, while *IAA8*, *IAA9*, *IAA13*, *IAA17*, *IAA27*, *ARF5*, and *ARF7* were reversed (Figure 7 and Supplementary Table 3). In agreement with previous reports, *IAA2* and *ARF9* upregulation expression (*IAA2*, Pascual et al., 2009; *IAA2*, Vriezen et al., 2008; *ARF9*, Serrani et al., 2008; *ARF9*, Wang et al., 2009), and *IAA9* and *ARF7* downregulated expression (*IAA9*, Wang et al., 2009; *ARF7*, de Jong et al., 2009) were good for the parthenocarpic fruit set (Figure 7 and Supplementary Table 3). Other *Aux/IAAs* and *ARFs* were inferred to be related to a parthenocarpic fruit set in tomato too. Validated by qRT-PCR, *LAX2* was significantly different at 0 DAA, and *GH3.1* was extremely significantly different between “R35-P” and “R35-N” at 0 and −2 DAA. *LAX2* related to IAA transport with a possible role in the regulation of auxin influx (Pattison and Catalá, 2012). As IAA-amido synthetase, GH3 can promote an IAA conjugation to amino acids to maintain auxin homeostasis (Molesini et al., 2020). In this study, *GH3.1* may be an important part in balancing the auxin synthesis and metabolism to ensure a fruit set in “R35-P” under any favorable or unprofitable conditions. Overall, these studies revealed that parthenocarpy can be achieved by manipulating auxin action at different levels, by acting on its biosynthesis, signaling cascade, and transport, corroborating the crucial role played by this hormone in the control of a fruit set (Molesini et al., 2020). Taken together, these results indicated that auxins played a pivotal role in controlling the fruit set of “R35-P.”

In natural parthenocarpic tomato *pat*, *pat-2*, and *pat3/pat4* mutants, higher GA levels have been found in the ovaries, and endogenous GA levels increase in tomato ovaries, following pollination (Mariotti et al., 2011; and the literature cited therein). These changes were not observed in our study. The levels of some active GAs, such as GA_1_, GA_3_, and GA_4_, were quite low, and GA_5_ and GA_7_ were under the detection limits at all-time points, although the GA_1_, GA_3_, and GA_4_ levels in Pe were higher than those in Ps and CK at most time points. Additionally, except for the content of GA_1_ in CK, the contents of GA_3_ and GA_4_ in Ps and CK were reduced, following pollination (after 0 DAA). It was evident that the expression of *GA20ox* genes, which leads to increased synthesis of GA20, the precursor of active GAs, is a key regulatory step in the GA biosynthetic pathway in *pat* mutants (Olimpieri et al., 2007) and in *pat-2* mutants (Fos et al., 2000). However, *GA20ox* genes were not found among the DEGs related to GAs in our transcriptomic study. This discrepancy between our results and previous investigations might be due to the difference in parthenocarpic mutant genes in “R35-P.” Fos et al. (2000) reported that there were significant differences in the levels and variation tendencies of GAs (except GA_20_) in ovaries in different tomato lines with different genetic backgrounds. Differences in the alteration of GA metabolism appeared in *pat-2* and *pat3/pat4* (Srivastava and Handa, 2005). Therefore, we inferred that the effects of GAs on the induction of a fruit set vary with the different parthenocarpic tomato sources. The application of exogenous auxins leads to parthenocarpic development with filled locules, while treatment with GAs leads to fruits with almost empty locular cavities (Serrani et al., 2007). The “R35-P” set parthenocarpic fruits with completely filled locules. Based on the findings described above, the parthenocarpy in “R35-P” might be independent of GA action.

In this study, a sustained gradual decrease in ABA content was found in Pe at all the studied periods, while ABA levels were the highest in the ovaries at anthesis (0 DAA) and dramatically declined after pollination/fertilization in Ps and CK. These results are consistent with previous findings (Srivastava and Handa, 2005; Mariotti et al., 2011). The ovary of tomato ceases to undergo cell division shortly (1–2 days) before anthesis and enters an “ovary arrest” state (Klap et al., 2017). ABA and ETH are hormones that inhibit plant growth. Large numbers of ABA and ETH genes were found in DEGs in hormone metabolism pathways at the transcript level, which were in agreement with the previous results (Vriezen et al., 2008). ABA and ETH concentrated highly before a fruit set, and then the expression of ETH and ABA was attenuated with pollination, suggesting that they are involved as antagonists of IAA and GAs to keep the pre-anthesis unpollinated ovary in a temporally protected and dormant state (Vriezen et al., 2008; Pascual et al., 2009). We also inferred that ABA and ETH may be involved as antagonists of IAA (IAA levels were the lowest at 0 DAA) to keep the pre-anthesis unpollinated ovary in a temporally protected and dormant state at anthesis. The ovary subsequently changes to an active state upon pollination and a fruit set in Ps and CK, while, in the parthenocarpic line, there is no state of “ovary arrest” at anthesis; therefore, the ABA content sustainably decreases in Pe. The differences accompanying this state led to the differential expression of CTK genes, which play a critical role in the stimulation of cell division. These results suggested that ABA and ETH may also play an important role in the regulation of a fruit set.

JA and SA are two major defense hormones found in response to wounding and defense against insect herbivores. Their roles in a tomato fruit set are not clearly understood. The higher level of JA in Pe may be caused by the wounding after removal of stigmas before anthesis. In addition, the variation tendency of JA differed among Pe, Ps, and CK. Therefore, JA may not be involved in the parthenocarpy of tomato. Unlike JA, the variation tendency of SA was similar in Ps and CK and lower than that in Pe at all four stages. We inferred that SA could respond to pollination/fertilization, and a higher content of SA is favorable to parthenocarpy in tomato.

Overall, high levels of IAA, GA_1_, and SA before anthesis and low levels of ABA and ETH were determined to be more conducive to the development of parthenocarpic tomato in this study. Auxins play a major role in a tomato fruit set (Vriezen et al., 2008; Pascual et al., 2009; Mariotti et al., 2011). Mariotti and others (2011) also suggested that elevated auxin levels in the ovaries initiate a cascade of events that finally promote fruit growth. These events include crosstalk with other hormones, especially the activation of GA metabolism, the increase in CTK, and the decrease in ABA levels (Mariotti et al., 2011). Consistent with these findings, we inferred that auxins govern the fate of fruit initiation events and play pivotal roles in integrating other endogenous hormone participation to promote a fruit set. Different parthenocarpic systems in tomato, such as normal parthenocarpic genotypes (Fos et al., 2000; Olimpieri et al., 2007; Pascual et al., 2009; Hazra et al., 2010; Takisawa et al., 2018, 2019; this study) or pollination-independent driven by hormonal treatment (Serrani et al., 2007, 2008; Vriezen et al., 2008; de Jong et al., 2009; Mariotti et al., 2011; Tang et al., 2015) or genetic engineering of phytohormone-related genes (Goetz et al., 2007; Marti et al., 2007; Martinelli et al., 2009; Molesini et al., 2009; Mazzucato et al., 2015; Mignolli et al., 2015) identified different pivotal phytohormones, and hormones-related genes showed differential expression in the fruit set and developing ovaries. In addition, the seedless fruits of these partenocarpic systems showed difference in fruit size or morphology or locular cavities and so on between the corresponding seeded fruits except with or without seeds. Collectively, this revealed that the pathway of the parthenocarpic fruit set and development might be varied in different parthenocarpic systems.

The earliest phase (up to anthesis) of fruit development involves the decision to abort or to continue with growth, and the molecular events observed in *pat-2* ovaries before anthesis, which are associated with parthenocarpic fruit growth, may modify the hormone content of the ovary before pollination (Fos et al., 2000). Consistent with this result, the difference in hormone contents mainly appeared at 0 DAA, and the DEGs present at the transcriptional level mainly appeared at −2 DAA in our study.

In this study, transcriptomic analyses showed that there were 7,479 DEGs at −2 DAA (Supplementary Table 4) and 2,378 DEGs (Supplementary Table 5) at 0 DAA among Pe, Ps, and CK, and the genes involved in hormone metabolism pathways composed only a fraction of those DEGs. Not only the complex hormonal regulatory network but also the interactions of many other genes controlled various aspects of the parthenocarpic fruit set. Our continuing studies on the mapping of major parthenocarpic QTLs to identify major parthenocarpic candidate genes and deeply analyze the transcriptome profile data may enable us to gain a better understanding of the flower-to-fruit transition mechanism of parthenocarpy in “R35-P.”

## Data Availability Statement

The original contributions presented in the study are publicly available. This data can be found here: NCBI repository, Accession number: PRJNA710539 (https://www.ncbi.nlm.nih.gov/bioproject/PRJNA710539).

## Author Contributions

SZ, JS, and WY conceived and designed the research. SZ, XG, ZH, LW, and LZ performed the experiments, conducted the fieldwork, and analyzed the data. SZ, XG, WY, HS, and LZ wrote the original manuscript. All the authors contributed to the article and critically revised the article.

## Conflict of Interest

The authors declare that the research was conducted in the absence of any commercial or financial relationships that could be construed as a potential conflict of interest.

## Publisher’s Note

All claims expressed in this article are solely those of the authors and do not necessarily represent those of their affiliated organizations, or those of the publisher, the editors and the reviewers. Any product that may be evaluated in this article, or claim that may be made by its manufacturer, is not guaranteed or endorsed by the publisher.

## Footnotes

1
http://ccb.jhu.edu/software/hisat2
